# Magnet-Retained Facial Prosthesis Combined with Maxillary Obturator

**DOI:** 10.1155/2013/406410

**Published:** 2013-05-08

**Authors:** Mahnaz Hatami, Hamid Badrian, Siamak Samanipoor, Marcelo Coelho Goiato

**Affiliations:** ^1^Department of Prosthodontics, School of Dentistry, Shahid Sadoughi University of Medical Sciences, Yazd, Iran; ^2^Dental Implant Research Center, School of Dentistry, Isfahan, Iran; ^3^Department of Endodontics, School of Dentistry, Shahid Bahonar University of Medical Sciences, Kerman, Iran; ^4^Faculty of Dentistry of Araçatuba, University of the State of São Paulo, Jose Bonifacio 1193, 16015-050 Araçatuba, SP, Brazil

## Abstract

Prosthetic rehabilitation of the midfacial defects has always perplexed prosthodontists. These defects lead to functional and esthetic deficiencies. The purpose of this clinical case report was the presentation of the prosthetic rehabilitation of an extraoral-intraoral defect using two-piece prosthesis magnetically connected. This prosthesis has dramatically improved the patient's speech, mastication, swallowing, and esthetic.

## 1. Introduction

Acquired midfacial defects may affect patients' speech, mastication, quality of life, psychology, and social behavior [1–4]. Midfacial defects are defined as defects in the middle third of the face in horizontal plane that communicate with intraoral maxillary defects. These defects can be classified into two major categories of midline and lateral-midline midfacial defects. Midline midfacial defects include complete or partial involvement of either nose or upper lip that communicate with an intraoral maxillary defect while the lateral midfacial defects include complete or partial involvement of cheek and orbital contents that communicate with an intraoral maxillary defect [5].

Midfacial defect can result from trauma, burns, most tumors of paranasal sinus, palatal epithelium, minor salivary glands [6–8], congenital abnormalities like vascular malformations [6], and some of other lesions like lethal midline granuloma [9] that require partial or radical maxillectomy. One of the other causes of such defects is mucormycosis that is caused by a fungus of the order Mucorales that is one of the most rapid fatal fungal infections known to man. Rhinocerebral mucormycosis is the most common type, and its extension to the orbit and brain is quite usual. The location of mucormycosis on the palate is rare and of late occurrence [10].

 Large midfacial defects are rarely rehabilitated by surgical reconstruction alone. They usually require a facial prosthesis to restore function and esthetic [11].

 In addition, an intraoral prosthesis such as an obturator should restore speech and deglutition. Fabrication of a facial prosthesis challenges the artistic ability of prosthodontists. On the other hand, size and weight of facial prostheses endanger the retention of them. This clinical case report describes the prosthodontic rehabilitation of an edentulous patient with a large midfacial (intraoral-extraoral) defect. The main aim of this rehabilitation was to provide the esthetic needs of the patient and to improve the patient's quality of life.

## 2. Case Report 

**Figure 1:**
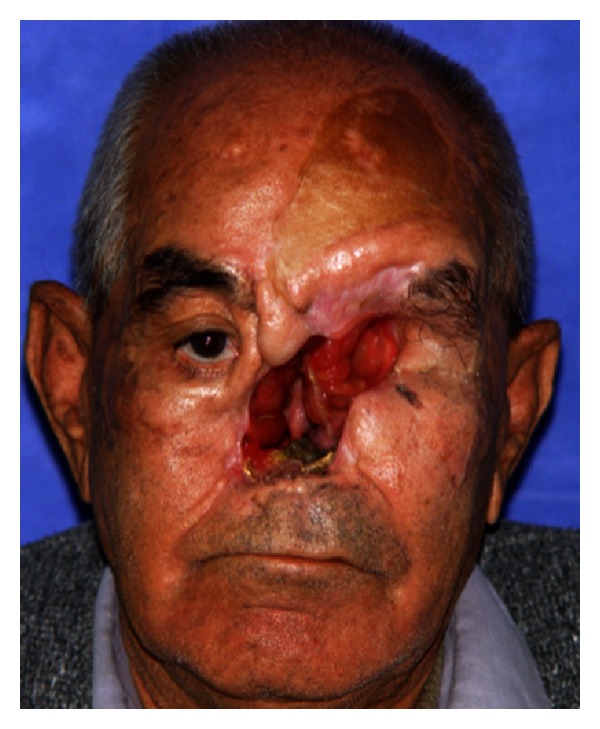
Midfacial defect after surgery.

**Figure 2:**
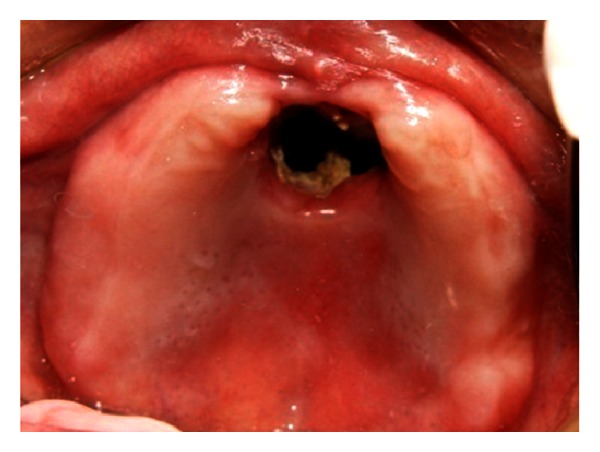
Intraoral view of maxillary defect.

 The patient was a 65-year-old edentulous male with a chief complaint of poor facial appearance and past medical history of diabetes mellitus. His facial tissues were affected by a fungal infection of rhinocerebral mucormycosis followed by diabetic ketoacidosis. For debridement and removal of necrotic tissues, ablative surgery has been done. Resected portions included anterior part of hard palate, nasal septum and conchae, left maxillary sinus, and orbital contents (Figures 1 and 2). Therefore, there was an open communication between the oral, nasal, and orbital cavities.

 After precise evaluation of the case, the proposed treatment plan was to construct a complete denture with obturator, as well as a facial prosthesis which would be attached to the obturator with cobalt samarium magnets (Jobmasters, Randallstown, USA).

 Treatment was started by the construction of a complete denture with obturator before facial prosthesis because anterior-posterior position of anterior denture teeth and labial flange has a basic role in lip support. The complete denture with obturator was made according to the procedure recommended by Zarb et al. [12] and Taylor [13].

**Figure 3:**
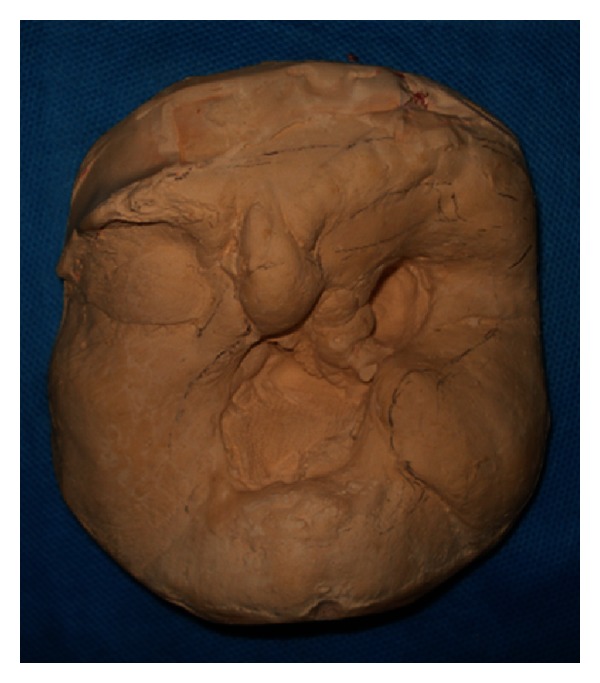
Working cast.

 The second stage was making an impression of the face defect and adjacent tissues using a thin layer of irreversible hydrocolloid (Hydrogum, Zhermack, Rovigo, Italy). The margin of impression was outlined on the face using the boxing wax (Kerr, Orange, CA, USA). Moist gauze was packed to prevent the flow of impression material into the undesired areas of the defect. The impression was reinforced with fast setting dental plaster (Ernst Hinrichs GMBH, Goslar, Germany) which has a thickness of 0.25 inch. The impression was boxed and poured in dental stone (Ernst Hinrichs GMBH, Goslar, Germany) (Figure 3).

**Figure 4:**
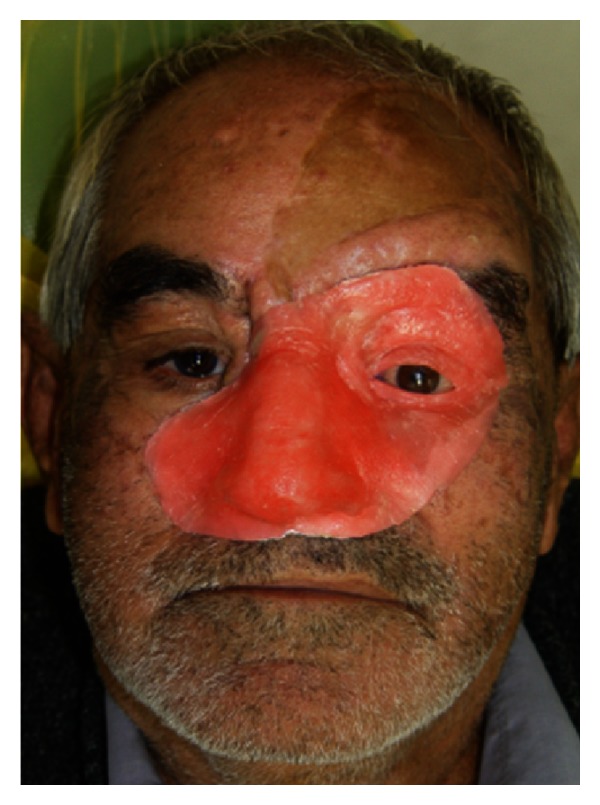
Wax pattern tryin.

 Then working cast was trimmed, defect undercuts were blocked out, and tinfoil substitute was applied to the cast. The waxing up of the nose, orbit, some portions of check and upper lip was developed on a thin acrylic resin baseplate (Acropars, Marlic Co., Tehran, Iran) which adapted on the master cast. Before this step, ocular prosthesis had been made using paper iris disk technique [13]. The cuplike pattern of orbital defect made the basis for inserting the ocular prosthesis within the defect in the same frontal, sagittal, and horizontal planes as the normal eye. The ocular prosthesis was fabricated into a position that matches the gaze of another normal eye when the patient was directly staring at a point at eye level at least 6 feet away. Then eyelid aperture was reproduced by softening and placing two small strips of wax over the ocular section. The shape of the lid opening should make the opening of the other normal eye [13] (Figure 4). 

**Figure 5:**
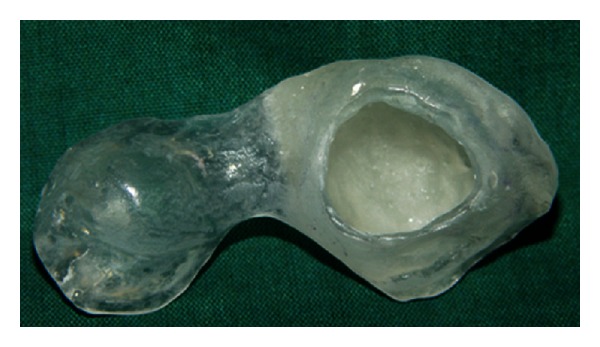
Hollow acrylic substructure.

 Patient's previous photographs and the references from his first circle relatives were taken as a guide for shaping the wax pattern. The contour of final surface and skin texture was fabricated by carving in lines and wrinkles which observed, by pressing a wet gauze square into softened wax. In the next visit, trial placement of wax pattern was performed on the patient's face and marginal discrepancies were refitted by corrective wax (Kerr, Orange, CA, USA). Then a hollow acrylic resin framework was made in posterior aspect of wax pattern for decreasing weight of the prosthesis [14] (Figure 5). 

 The wax pattern was flask using die stone (Ernst Hinrichs GMBH, Goslar, Germany) to form a mold for packing the silicone. Wax elimination was performed in usual manner [15]. Then, the acrylic substructure was placed on the mold and was packed with a MDX4-4210-base silicone (Dow Corning Corp., Midland, USA). Laminar intrinsic staining was used in packing according to the patient's skin color [13]. The silicone was heated for 2 hours at 90°C, disinvested, trimmed, and cleaned. The prosthesis was trial fitted and extrinsically colored by medical adhesive type A (Dow corning, Midland, MI) and oil pigments (Factor *ΙΙ*, Lakeside, USA).

 Autopolymerizing resin (Acropars, Marlic Co., Tehran, Iran) was initially used to attach a magnet on the superior aspect of the obturator. Indelible pencil was drawn on the surface of the first magnet, and the facial prosthesis was positioned in its location to demarcate the area of most contact.

 At the time of insertion of the facial prosthesis, the patient was instructed to close in maximum intercuspal position with obturator placed in the mouth. 

**Figure 6:**
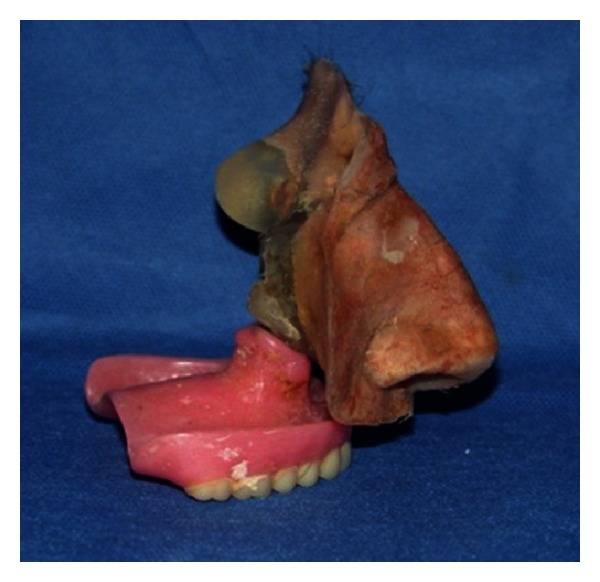
Attached intra and extraoral prostheses.

**Figure 7:**
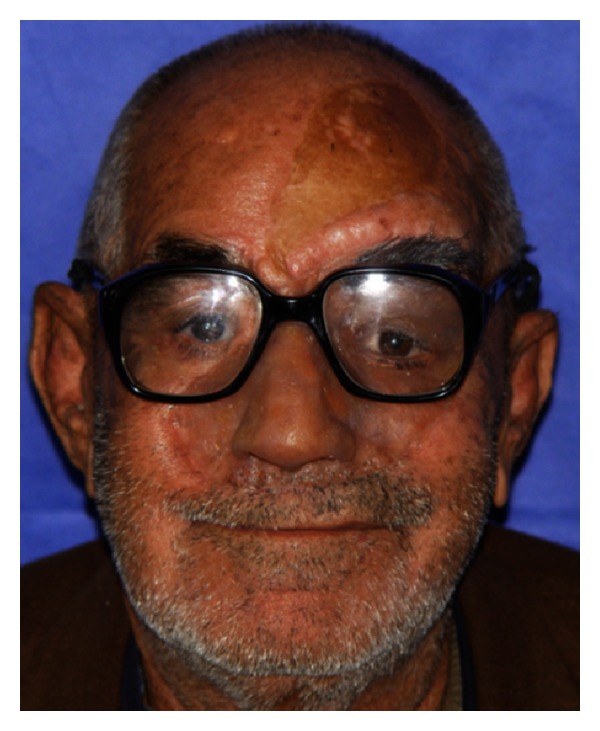
Completed facial prosthesis in place.

 On the demarcate area of the facial prosthesis extension (acrylic substructure), the second magnet was attached to self-curing acrylic resin. The extraoral prosthesis had adequate retention after using magnets and eyeglasses (Figures 6 and 7). Extra support for the glasses was gained by attaching an elastic band around the back of the head from one earpiece to another one.

 The patient was given hygiene instructions for cleaning both prostheses. The patient attended recall visits every 4 to 5 months. During two years after prosthesis insertion, the prosthesis was still serviceable and the patient was pleased.

 An esthetic improvement, intelligible speaking, andimproved deglutition and mastication were achieved for the patient by this prosthetic reconstruction.

## 3. Discussion

 Restoration of midfacial defects can be accomplished surgically or prosthetically or by using a combination of both methods. Selection of each method depends on many factors including size, location of the defect, and age of patient [13].

 Acceptable esthetic results usually can be obtained by a facial prosthesis. However, retention of a large prosthesis can be challenging.

 Various methods of auxiliary retention for facial prosthesis have been described in the literature; they include eyeglasses [16], denture extensions that engage tissue undercuts [16, 17], magnets [16, 18], facial prosthetic adhesives [16], or combination of the above [16, 17, 19], and craniofacial implants [16, 17, 20, 21]. Respiratory epithelium is easily traumatized by frictional contact with prosthesis and limits the use of anatomic undercuts [22]. Soft tissues around defects may not always be ideal for adhesive retention because movements that occur during smiling compromise adaptation of prosthesis margins [23, 24].

 For the first time, Nadeau [25] described the use of combination of extra- and intraoral prostheses connected by magnets. Durability of surface coatings of the long-term magnets is a major concern; hence, it is advised to use the magnets with strong surface coatings.

 Connecting these prostheses often results in movement of facial prosthesis during mastication [26, 27]. The use of eyeglasses alone for retaining a nasal prosthesis has been well documented [28–30]. Although craniofacial implants may provide the most reliable prosthesis retention, additional surgeries, expenses, inadequate quantity or quality of the bone, and prior radiation to the area may contraindicate this type of treatment [31, 32].

 A hollow acrylic resin framework used for facial prosthesis is advantageous as there is no need to fabricate the whole prosthesis again in case of discoloration or damage of the silicone layer because the outer silicon layer can be removed and repacked with the new silicon on the acrylic resin framework if the mold is preserved.

 The advantages of this prosthesis are that the technique is noninvasive, cost effective, tissue tolerant, esthetic, comfortable to use, and easy to clean. The difficulty in maxillofacial rehabilitation of large defects often involves the compromise of functional adequacy versus esthetic.

## 4. Conclusion

 Satisfactory functional and esthetic results are achievable in patients with a large lateral midfacial defect using a hollow acrylic resin framework for silicon facial prosthesis. Retention of facial prosthesis can be satisfactorily achieved with the use of strong magnets provided that the facial prosthesis is light in weight.
